# Epidemiology of human papillomavirus infection in women from Xiamen, China, 2013 to 2023

**DOI:** 10.3389/fpubh.2024.1332696

**Published:** 2024-03-21

**Authors:** Xingmei Yao, Qing Li, Yu Chen, Zhuowen Du, Yanru Huang, Yixi Zhou, Jian Zhang, Wenbo Wang, Lutan Zhang, Jieqiong Xie, Chao Xu, Yunsheng Ge, Yulin Zhou

**Affiliations:** ^1^Department of Central Laboratory, Women and Children’s Hospital, School of Medicine, Xiamen University, Xiamen, Fujian, China; ^2^Department of Women Health Care, Women and Children’s Hospital, School of Medicine, Xiamen University, Xiamen, Fujian, China; ^3^United Diagnostic and Research Center for Clinical Genetics, Women and Children’s Hospital, School of Medicine and School of Public Health, Xiamen University, Xiamen, Fujian, China; ^4^Department of Information Center, Women and Children’s Hospital, School of Medicine, Xiamen University, Xiamen, Fujian, China

**Keywords:** human papillomavirus (HPV), infection, genotype, prevalence, cervical cancer

## Abstract

**Background:**

Cervical cancer is primarily caused by HPV infection. The epidemiology of HPV infection in specific areas is of great meaning of guide cervical cancer screening and formulating HPV vaccination strategies. Here, we evaluated the epidemiological characteristics of HPV infection in Xiamen population.

**Methods:**

In total, 159,049 cervical exfoliated cell samples collected from female outpatients in Women and Children’s Hospital, School of Medicine, Xiamen between January 2013 and July 2023 were analyzed. HPV DNA detection was performed using HPV genotyping kits (Hybribio Limited Corp, China). An analysis was conducted on the prevalence of HPV infection, taking into account factors such as age, year, and multiple patterns of HPV infection. The differences in prevalence among age groups and years were compared using χ^2^ test.

**Results:**

The overall prevalence of any 21 HPV genotypes was 18.4%, of which the high-risk HPV (HR-HPV) positive rate was 14.6%. The age-specific prevalence of HPV infection showed a bimodal distribution, with two distinct peaks, one at <25 years (31.2%) and the other at 60–64 years (32.9%). There was a downward trend in the prevalence of HPV infection over time, decreasing from 26.2% in 2013 to 14.5% in 2021, and then increasing to 19.0% in 2023. The five most prevent HR-HPV genotypes were HPV52 (4.0%), 58 (2.6%), 16 (2.5%), 51 (1.8%), and 39 (1.7%). Among the positive cases, 76.7% were detected with only one genotype and 23.3% with multiple genotypes. The most common co-infection was HPV52 + HPV58 (0.24%), followed by HPV16 + HPV52 (0.24%), HPV52 + HPV53 (0.21%), HPV52 + HPV81 (0.21%), HPV51 + HPV52 (0.19%), HPV16 + HPV58 (0.18%), and HPV39 + HPV52 (0.17%).

**Conclusion:**

The study provided the largest scale information on the recent epidemiological characteristics of HPV infection in Xiamen, and even in Fujian Province, China, which would support making the prevention and control strategies for cervical cancer in the region.

## Introduction

Cervical cancer is the fourth most prevalent cancer among women worldwide (1). More than 85% of cervical cancer cases and deaths occur in developing countries, such as China (2). In recent years, the incidence and mortality of cervical cancer in China have been increasing. An estimated number of 109,741 new cases and 59,060 deaths from cervical cancer were recorded annually in China (3), accounting for 20% of the annual global incidence (604,127 new cases) and 17% of the annual global mortality (341,831 deaths) (1). Therefore, it is urgent to take effective prevention and control measures to reduce the burden of cervical cancer in China.

It is widely acknowledged that Human papillomavirus (HPV) infection is an etiological factor for cervical cancer (4, 5). HPV is a common sexually transmitted pathogenic microorganism. More than 200 HPV genotypes have been identified to date (6). Of which, approximately 40 genotypes can infect genital tract and cause related lesions, and they are categorized as high-risk (HR-HPV) and low-risk (LR-HPV) according to their potential carcinogenicity in humans (7, 8). The HR-HPV include HPV16, 18, 31, 33, 35, 39, 45, 51, 52, 56, 58, 59, 68, and it has been suggested that there was high variation in potential carcinogenicity among these genotypes (9). HPV16 and 18, the most carcinogenic genotypes, cause about 70% of cervical cancers around the world (1). Whereas, LR-HPV, such as HPV6 and HPV11, may cause genital warts and some other hyperplastic lesions.

The prophylactic HPV vaccine is the most effective primary prevention and control measure for cervical cancer or other HPV-related diseases. Currently, there are 6 licensed prophylactic HPV vaccines, including three bivalent vaccines (HPV16/18, Cervarix®, Cecolin®, and Walrinvax®), two quadrivalent vaccines (HPV6/11/16/18, Gardasil®9 and Cervavax®), and one nonavalent vaccine (HPV6/11/16/18/31/33/45/52/58, Gardasil® 9) (10). The HPV vaccine has been widely used worldwide and has shown a significant effect. A significant decrease of vaccine-type related high-grade cervical lesions and cervical cancer has been observed in countries with high HPV vaccine coverage. However, HPV vaccines showed prominently genotype restricted efficacy, which can only prevent vaccine-type infection and vaccine-type related lesions. It has been reported that the prevalence and distribution of HPV genotypes varied quite a lot across geographic regions (1), and even varied among regions within the same country (11). Therefore, investigating the epidemiological characteristics of HPV infection in a certain population is the foundation of making HPV vaccination strategies in this area.

In addition, the prophylactic HPV vaccine cannot clear the acquired infection, and the current HPV vaccines do not cover all high-risk genotypes, cervical cancer screening should still be performed even after HPV vaccination. Due to the important role of HPV infection in the development of cervical cancer and high-sensitive, objective, and repeatability of HPV DNA detection, the role of HPV DNA detection has been constantly improved in cervical cancer screening. It is important to note that HPV DNA detection was recommended as the preferred screening method for cervical cancer screening by the WHO guideline (12). The implementation of HPV screening, particularly for HR-HPV, has the potential to decrease the risk of cervical cancer. Hence, it is essential to update information on the prevalence of type-specific HPV and its distribution in different geographic regions for regional HPV screening strategies.

Xiamen, an economically developed city in Fujian province, is situated in southeast China. To be our knowledge, a population-based study on the epidemiology of HPV infection is limited and outdated. Our colleagues Shen et al. (13) have previously investigated the prevalence and distribution of HPV using the data collected from November 2019 to June 2021, however, the study period coincided with the COVID-19 epidemic period, which may have impact on the characteristics of HPV. Thus, to confirm the results and to obtain additional information, in the study, we conducted a larger sample size study over a long period of time to investigate the epidemiological characteristics of HPV infections, which would support making regional prevention and control strategies for cervical cancer in Xiamen, China. In addition, from September 2020, adolescent girls aged 13.5 to 14.5 years in Xiamen were vaccinated free with HPV bivalent vaccine (Cecolin^®^), and the free cervical cancer screening program for women aged 35–64 years was implemented in Xiamen from 2022, our results would provide baseline information for estimating effect of HPV vaccination and HPV screening in this region.

## Methods

### Study population

The study population consisted of women who underwent HPV testing in gynecological outpatient and health examination center of Women and Children’s Hospital, School of Medicine, Xiamen University, China from January 2013 to July 2023. All women met following conditions before sampling: (1) no sexual intercourse in 48 h; (2) no intravaginal medication in 48 h; (3) during a non-menstrual period. Women with available HPV genotyping results were included in the study, the exclusion criteria were pregnancy, incomplete identity information and baseline information (e.g., age, report date). This study was approved by the Ethics Committee of Women and Children’s Hospital, School of Medicine, Xiamen University (approval number KY-2023-073-H01).

### Sample collection and HPV DNA detection

Gynecologists collected cervical exfoliated cell samples from outpatients according to the established operating procedure, and stored the samples in preservation solution for HPV DNA testing. HPV DNA typing was performed using one of the two commercial HPV GenoArray Diagnostic kits, HBGA-21PKG and HBGA-37PKG (Hybribio Limited Corp, Chaozhou, Guangdong, China), which were based on DNA amplification with HPV L1 consensus polymerase chain reaction primers, can detect 21 and 37 HPV genotypes, respectively. The 21 genotypes kit was used from 2013 to 2017, which detects 13 HR-HPV genotypes (16, 18, 31, 33, 35, 39, 45, 51, 52, 56, 58, 59, and 68) and 8 LR-HPV genotypes (6, 11, 42, 43, 44, 53, 66, and 81). Since 2018, the upgraded version, the 37 genotypes kit was used, which can detect other 16 genotypes, including HPV26, 34, 40, 54, 55, 57, 61, 67, 69, 70, 71, 72, 73, 82, 83, 84. These two kits have been approved by the National Medical Products Administration (NMPA). The tests were conducted strictly in accordance with the manufacturer’s instruction. In brief, the main points of the experimental protocol are as follows: (1) DNA extraction: DNA was extracted based on magnetic beads method. The principle of the method is that the powerful protein denaturant in magnetic bead binding solution dissolve the protein and dissociate the DNA, the released DNA is bound to the magnetic bead, and then the impurities are removed by the magnetic bead washing solution, and the pure DNA is eluted down by the eluent. DNA extraction was performed by an automated nucleic acid extraction instrument using a pre-packed nucleic acid extraction kit (DaAn Gene Co, Ltd., China). (2) PCR amplification: the extracted DNA was subject to PCR amplification using HPV L1 consensus primers, the amplification reagent was configured according to the PCR mixture of 23.25 μL, DNA polymerase 0.75 μL and DNA 1 μL per sample. Amplification was started with initial denaturation at 20°C for 10 min and then 95°C for 9 min, followed by 40 cycles of 95°C for 20s, 55°C for 30s, 72°C for 30s and a final extension at 72°C for 5 min. (3) hybridization: the 25 μL of PCR products was further examined by flow through hybridization using HPV GenoArray Diagnostic Kit, the specific detection steps were as follows: 25 μL of PCR products were denatured by heating at 95°C for 5 min and then bathed in ice for 2 min, mixed with 0.5 mL pre-warmed hybridization solution and incubated for 10 min, after which a blocking solution was added. This flow-through hybridization procedure was performed in a sample well atop a Hybrimem HPV-21/ HPV-37 membrane containing immobilized probes against which target molecules were directed. Streptavidin-horseradish peroxidase conjugate was added to bind to the biotinylated PCR products. The direct visualization of the breakdown product (purple dot) of the substrate nitroblue tetrazolium-5-bromo-4-chloro3-indolylphosphate was interpreted as positive for the corresponding HPV DNA type as indicated on the schematic diagram of the membrane provided with the test kit. HPV negative and positive controls provided in the kit were simultaneously detected in every test for quality control (13–15).

### Statistical analysis

The first sample for HPV genotype evaluation from each woman collected between January 2013 and July 2023 was considered, and therefore ensured that women were only included once in the analysis. In the study, the common 21 genotypes detected by both HBGA-21PKG and HBGA-37PKG were analyzed. We calculated the overall, age-specific, and year-specific prevalence of HPV infection, respectively. The difference in the prevalence of HPV infection among age groups and year were analyzed using χ^2^ test. The age-specific infection pattern (single, double and multiple infection) of HPV genotypes was also evaluated. Subsequently, we calculated the prevalence of single and multiple infection, respectively. Moreover, to quantify the co-infection preference of 21 HPV genotypes, we also created a heatmap of prevalence of any two HPV genotypes. *p* < 0.05 was considered statistically significant. Statistical analyses were performed using SAS version 9.4 software (SAS Institute, Cary, North Carolina).

## Results

### HPV prevalence

**Figure 1:**
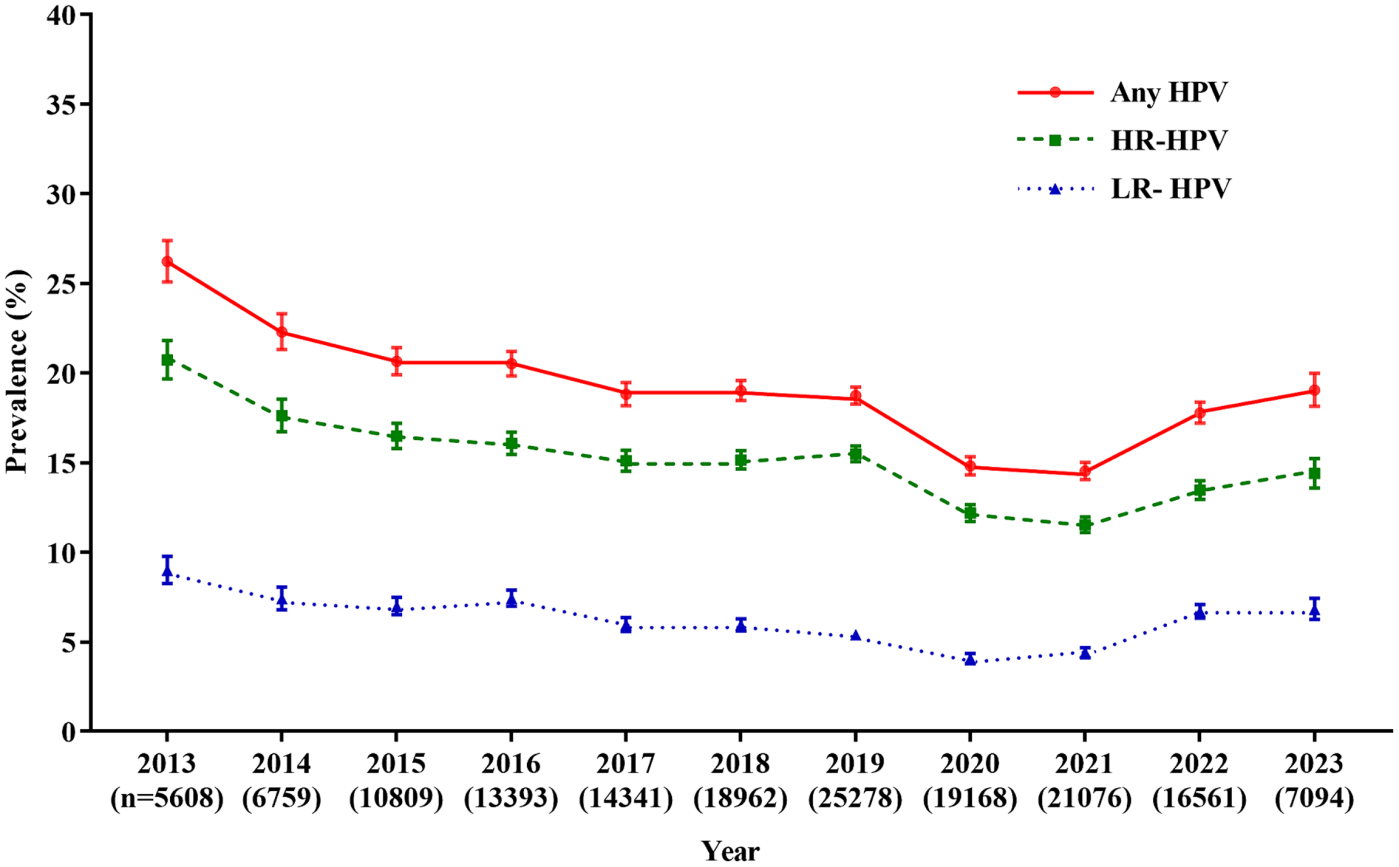
Prevalence of HPV infection over the past 10.5 years. Any HPV: any of 21 detected HPV types; HR-HPV, high-risk HPV types (16, 18, 31, 33, 35, 39, 45, 51, 52, 56, 58, 59, and 68); LR-HPV, low-risk HPV types (6, 11, 42, 43, 44, 53, 66, and 81); HPV, human papillomavirus.

From January 2013 to July 2023, a total of 159,049 female outpatients aged 15 to 92 (mean age: 35.9 ± 9.2) years old were involved in the study. Of the 159,049 specimens, 29,198 women were positive for HPV, with an overall HPV infection rate of 18.4% (29,198/159,049). The prevalence of HR-HPV and LR-HPV were 14.6% (23,252/159,049) and 5.9% (9,399/159,049), respectively. The prevalence of overall HPV infection showed a downward trend over time (Cochran-Armitage χ^2^ = 21.03, *p* < 0.0001), the overall prevalence of HPV infection decreased from 26.2% (1,471/5,608) in 2013 to 14.5% (3,063/21,076) in 2021, and then increased to 19.0% (1,351/7,094) in 2023. Similar trends were observed for the HR-HPV (decreased from 20.7% in 2013 to 11.5% in 2021, and then increased to 14.4% in 2023) and LR-HPV (decreased from 9.0% in 2013 to 4.4% in 2021, and then increased to 6.8% in 2023; Figure 1; Supplementary Table S1).

**Figure 2:**
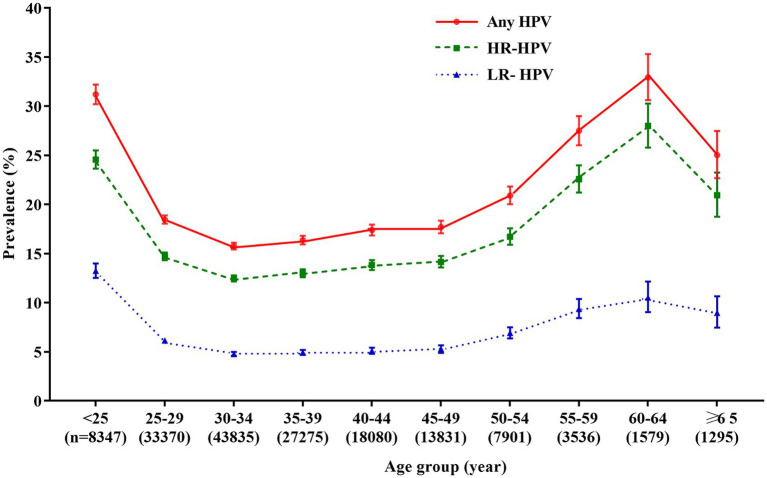
Age-specific prevalence of HPV infection. Any HPV: any of 21 detected HPV types; HR-HPV, high-risk HPV types (16, 18, 31, 33, 35, 39, 45, 51, 52, 56, 58, 59, and 68); LR-HPV, low-risk HPV types (6, 11, 42, 43, 44, 53, 66, and 81); HPV, human papillomavirus.

When women were stratified into 10 age groups of <25 years, 25–29 years, 30–34 years, 35–39 years, 40–44 years, 45–49 years, 50–54 years, 55–59 years, 60–64 years, and ≥ 65 years, we found that the prevalence was significantly different (χ^2^ = 1697.20, *p* < 0.0001), and the age-specific prevalence of HPV infection was “U-shaped” distribution, with the highest overall prevalence of HPV was found in women aged 60–64 years (32.9%, 520/1,579), while the lowest HPV prevalence was found in women aged 30–34 years (15.8%, 6,910/43,835). Similarly, the prevalence of HR-HPV and LR-HPV also differed among these age groups (*p* < 0.0001). The highest prevalences of HR-HPV and LR-HPV were found in women aged 60–64 years (28.0%, 442/1,579) and women <25 years (13.2%, 1,105/8,347), respectively. While the lowest rates were both found in women aged 30–34 years (HR-HPV: 12.5%, 5,466/43,835; LR-HPV: 4.8%, 2,090/43,835; Figure 2; Supplementary Table S2).

### Single and multiple HPV infections

Single HPV infection was observed to be the most common pattern, accounting for 76.7% (22,407/29,198) of all HPV positive cases, with an overall prevalence of 14.1% (22,407/159,049). The prevalence of multiple infections was relatively low (4.3%, 6,791/159,049), accounting for 23.3% (6,791/29,198) of HPV positive cases. And of which, most women were infected with 2 genotypes, with a prevalence of 3.2% (5,006/159,049), accounting for 17.1% (5,006/29,198) of HPV positive cases. Three or more genotypes were found in 1,785 women with a prevalence of 1.1% (1,785/159,049), accounting for 6.1% (1,785/29,198) of HPV positive cases. We further analyzed multiple infection cases with HR-HPV types only, LR-HPV types only, and combination of HR-HPV and LR-HPV types. HR-HPV only infection accounted for 44.6% (3,030/6,791) of multiple infections, with a prevalence of 1.9% (3,030/159,049). 50.8% (3,453/6,791) of multiple infections showed both HR-HPV and LR-HPV infections for a prevalence of 2.2% (3,453/159,049) for all tested women. While only 308 women had 2 or more LR-HPV types, accounting for 4.5% (308/6,791) of multiple infections. The prevalences of multiple infections were also different among age groups, however, the pattern was similar, co-infection with both HR-HPV and LR-HPV was most common in all age groups (Table 1).

**Table 1:** Prevalence of HPV single/multiple infection in different age groups.

Age group	<25 (*N* = 8,347)	25–29 (*N* = 33,370)	30–34 (*N* = 43,835)	35–39 (*N* = 27,275)	40–44 (*N* = 18,080)	45–49 (*N* = 13,831)	50–54 (*N* = 7,901)	55–59 (*N* = 3,536)	60–64 (*N* = 1,579)	≥65 (*n* = 1,295)	Total (*N =* 159,049)
Age, years, mean (SD)	22.6 (1.5)	27.3 (1.4)	31.8 (1.4)	36.8 (1.4)	41.9 (1.4)	46.8 (1.4)	51.7 (1.4)	56.7 (1.4)	61.7 (1.4)	69.8 (5.1)	35.9 (9.2)
Positive for HPV Infection, n (%)											
Single infection	1,651 (19.8)	4,608 (13.8)	5,520 (12.6)	3,582 (13.1)	2,570 (14.2)	1964 (14.2)	1,270 (16.1)	689 (19.5)	349 (22.1)	204 (15.8)	22,407 (14.1)
multiple infections	954 (11.4)	1,551 (4.6)	1,390 (3.2)	883 (3.2)	574 (3.2)	483 (3.5)	382 (4.8)	283 (8.0)	171 (10.8)	120 (9.3)	6,791 (4.3)
2 infections	606 (7.3)	1,128 (3.4)	1,078 (2.5)	701 (2.6)	457 (2.5)	388 (2.8)	283 (3.6)	185 (5.2)	117 (7.4)	63 (4.9)	5,006 (3.2)
≥3 infections	348 (4.2)	423 (1.3)	312 (0.7)	182 (0.7)	117 (0.7)	95 (0.7)	99 (1.3)	98 (2.8)	54 (3.4)	57 (4.4)	1785 (1.1)
Pattern of multiple Infections, n (%)											
HR-HPV + HR-HPV	355 (4.3)	683 (2.0)	672 (1.5)	420 (1.5)	277 (1.5)	224 (1.6)	157 (2.0)	118 (3.3)	73 (4.6)	51 (3.9)	3,030 (1.9)
HR-HPV + LR-HPV	550 (6.6)	801 (2.4)	646 (1.5)	420 (1.5)	275 (1.5)	239 (1.7)	214 (2.7)	157 (4.4)	88 (5.6)	63 (4.9)	3,453 (2.2)
LR-HPV + LR-HPV	49 (0.6)	67 (0.2)	72 (0.2)	43 (0.2)	22 (0.1)	20 (0.1)	11 (0.1)	8 (0.2)	10 (0.6)	6 (0.5)	308 (0.2)

HR-HPV, high-risk HPV types (16, 18, 31, 33, 35, 39, 45, 51, 52, 56, 58, 59, and 68); LR-HPV, low-risk HPV types (6, 11, 42, 43, 44, 53, 66, and 81); HPV, human papillomavirus.

### HPV genotype distribution

**Figure 3:**
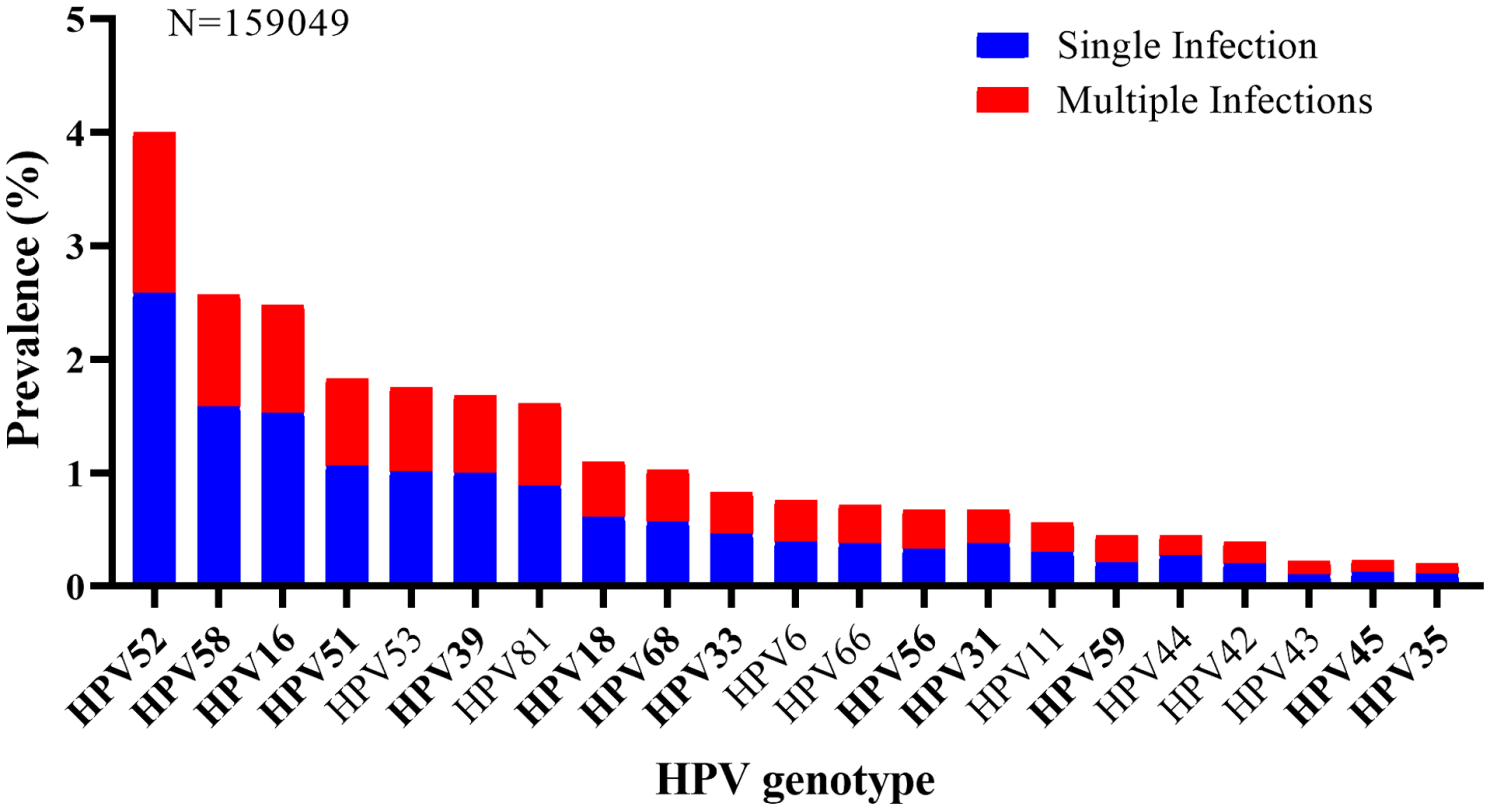
HPV genotypes distribution of single (in blue) and multiple infections (in red).

As shown in Figure 3, HPV52 (4.0%, 6,358/159,049) was the most commonly detected genotypes, followed by HPV58 (2.6%, 4,097/159,049), 16 (2.5%, 3,958/159,049), 51 (1.8%, 2,905/159,049), and 53 (1.7%, 2,772/159,049). And other genotypes with a prevalence of more than 1.0% included HPV39 (1.7%), 81 (1.6%), 18 (1.1%), and 68 (1.0%). Additionally, the five most prevalent HPV genotypes also accounted for the highest rates in the top five of both single and multiple infections. In single infection, the prevalences of the top five prevalent genotypes were HPV52 (2.6%, 4,099/159,049), HPV58 (1.6%, 2,520/159,049), HPV16 (1.5%, 2,438/159,049), HPV51 (1.1%, 1,678/159,049), and HPV53 (1.0%, 1,601/159,049), and that were HPV52 (1.4%, 2,259/159,049), HPV58 (1.0%, 1,577/159,049), HPV16 (1.0%, 1,520/159,049), HPV51 (0.8%, 1,227/159,049), and HPV53 (0.7%, 1,171/159,049) in multiple infections.

**Figure 4:**
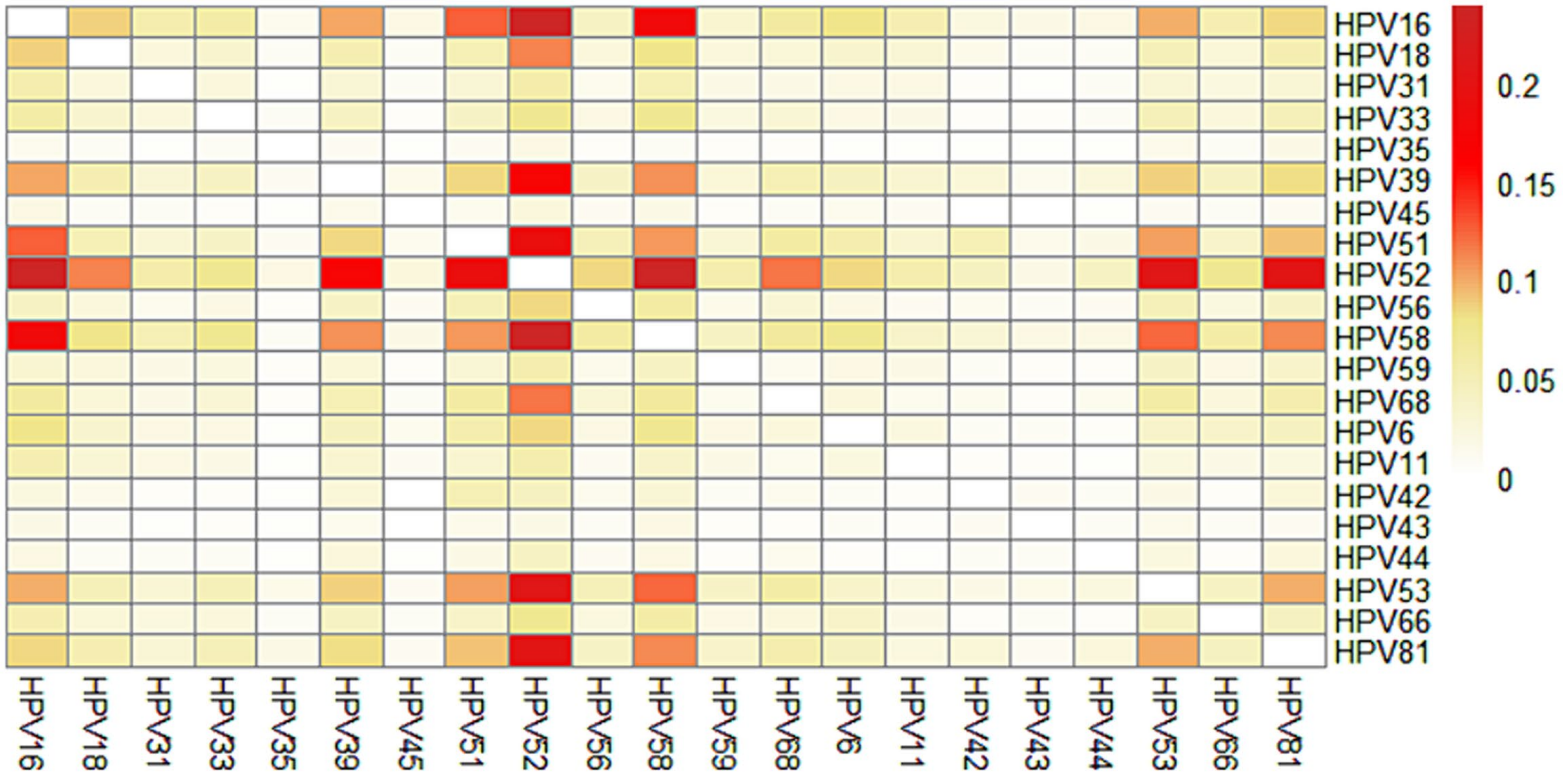
Prevalence of HPV involved in women with HPV co-infection by genotypes.

We further calculated the prevalence of co-infection with any 2 genotypes out of the 21 HPV genotypes. The most common co-infection was HPV52 + HPV58 (0.24%), followed by HPV16 + HPV52 (0.24%), HPV52 + HPV53 (0.21%), HPV52 + HPV81 (0.21%), HPV51 + HPV52 (0.19%), HPV16 + HPV58 (0.18%), HPV39 + HPV52 (0.17%). Interestingly, we found that HPV35, HPV45, HPV43, HPV44 were rarely co-infected with other genotypes (Figure 4; Supplementary Table S3).

## Discussion

Epidemiological characteristics of HPV infection in a region are one of the important steps toward cervical cancer prevention. Here, we conducted a large clinic-base study to investigate the characteristics of HPV infection in Xiamen, southeast China. The study population was composed of gynecological outpatients and asymptomatic women, which would be more representative of the overall HPV infection in women from Xiamen.

The prevalence of HPV infection varied considerably across geographic regions. Globally, the highest prevalence of HPV infection was in some countries in Africa, South America and Europe (≥33.87%), followed by some European countries and Australia (16.93–33.87%), and some countries in North America and South America, and China (<16.93%) (1). Even in one country, especially in China, the prevalence of HPV infection varies greatly due to its large population and territory. In the current study, the prevalence of HPV was 18.4%, which was higher than that reported in a pooled analysis in China (15.5%) (16), but lower than that in Guangzhou (21.7%) (17), Zhejiang (22.3%) (18), Jiangxi (22.5%) (19), and other northern regions, such as Heilongjiang (27.1%) (20) and Henan (19.7%) (21). Significantly, the prevalence of HPV was also much lower than that in a previous study in Fuzhou (38.3%) (22). The reported prevalence varies among studies because of the difference in population composition, testing methods, and region. In addition, the sampling periods may be part of the reason. We found the prevalence of overall HPV, HR-HPV and LR-HPV showed a downward trend from 2013 to 2021 (overall HPV: from 26.2 to 14.5%, HR-HPV: from 20.7 to 11.5%, LR-HPV: from 9.0 to 4.4%). Similarly, the overall HPV prevalence was found to decline from 28.7% in 2011 to 17.8% in 2014 in Zhejiang (23). The reason for the decline in HPV prevalence may be due to the improvement of health awareness and the popularization of HPV. Considering an increasing number of asymptomatic women attended HPV screening, the prevalence of HPV decreased, and tended to be natural infection rate. Interestingly, the prevalence of HPV increased in 2022 and 2023, which may be attributed to the free cervical cancer screening program implemented in Xiamen from 2022. The data of free cervical cancer screening program was not included in this study, participation of asymptomatic women in cervical cancer screening programs has led to an increase in the proportion of gynecological outpatients in our study population, which in turn contributed to the increase in HPV infection rates. However, the reasons for the increase of HPV infection in these 2 years remain unknown and should be investigated. The changes of HPV infection should be monitored continuously in Xiamen.

The prevalence of HPV infection was also different among age groups. Globally, the prevalence of HPV infection peaks (~30%) shortly after the debut of sexual activity (<25 years), and then quickly decreases and keeps at low rate (<10%) in women aged 35–44 years, and then increases slightly in women aged ≥65 years (1). However, numerous studies have demonstrated that the age-specific prevalence of HPV showed a bimodal distribution in Chinese women (23–28). In line with previous studies, our study found that the age-specific prevalences of overall HPV, HR-HPV, and LR-HPV all showed a bimodal distribution, with a peak at the women <25 years and another at the women aged 60–64 years. Although the age of infection peak varied in different studies, the first peak was generally found in young women with early twenties, and the second peak was usually found in menopausal women. The first peak observed in young women may be related to sexual activity and immature immunity to HPV. It is reported that the risk of acquiring HPV infection was 46% at 3 years after first intercourse, and the median time from first intercourse to first detection of HPV was only 2.6 months (29). Thus, considering the nature of prophylactic HPV vaccines, it is the optimum time for adolescent females to receive HPV vaccines before the onset of sexual activity (i.e., before exposure to HPV). The understanding of the second peak in older women was limited, HPV persistence or the reactivation of potential HPV caused by physiological and immune disorders during the period of menopause, and changes in the sexual behavior during middle age may be the explanations (30). Therefore, it is necessary to conduct HPV screening in perimenopausal women in China.

According to HPV prevalence surveys, HPV16 and 18 are the most prevalent genotypes worldwide, which cause approximately 70% of cervical cancer (1). However, it was reported that HPV52 and HPV58 are more prevalent in Asian, especially in China (31–33). In our study, HPV52, 58, 16, 51 and 53 were found to be the five most prevalent HPV genotypes in Xiamen, which was similar to previous surveys in Sichuan and Yunnan province. However, in a previous study conducted in Fujian, HPV16 was identified to be the most frequent (8.6%) (22). This might be due to the difference in population composition and survey period. It has been reported that HPV52 and 58 account for 33.3% of high-grade lesions and 14.7% of cervical cancer, and HPV51 accounts for 3.9% of high-grade lesions in China (1). Hao et al. (34) suggested that HPV51 and HPV53 were found in 6.3% of HPV infection and 8.9% of cervical cancer in Jilin province, China. Considering the high prevalence and carcinogenicity of HPV52, 58, 51 and 53, except for HPV16 and 18, more attention also should be paid to preventing and control these genotypes infection in Xiamen, including the development of HPV prophylactic vaccines based on HPV16 and HPV18, covering HPV52, 58, 51 and 53, and implementing the HPV screening programs which focus on HPV52, 58, 51 and 53, as with HPV16 and 18.

Co-infection with multiple genotypes is common in HPV-positive individuals. In our study, multiple infections accounted for 23.3% of all infections, which is lower than that in Guangzhou (26.5%) (17), Shanghai (36.6%) and Beijing (27.7%), and similar to that in Zhejiang (22.5%) and Shanxi (24.3%) (35). There is ongoing debate regarding the impact of multiple HPV infections on the development of cervical cancer. Several studies have suggested an extended duration of multiple HPV infections and a stronger association between multiple HPV infections and precancerous lesions/cervical cancer (36–39). However, some other studies have reported that multiple HPV infections were no additive or synergistic effect on the development of cervical precancerous lesions and cervical cancer (40, 41). The carcinogenicity of multiple infections may be related to the different HPV genotypes combinations. However, few studies focus on whether different HPV genotypes combinations could interact on promoting or decreasing the oncogenicity. Carrillo-Garcia et al. (38) found that the co-infection of 68 with 16 increases the risk of ICC/HISL. Wang et al. (39) suggested that multiple HR-HPV infection with HPV16/18 had a higher risk of CIN2+, while multiple HR-HPV infection without HPV16/18 did not increase the risk significantly. Brun et al. (42) also found it is probable that only specific combinations of HPV can be associated with a clinically significant impact. In the study, we found that HPV52 + HPV58 and HPV16 + HPV52 were the most prevalent combinations of genotypes, which is consistent with the findings of Wang et al. (43), while Chen et al. (23) reported that HPV16 + HPV58, HPV16 + HPV18 and HPV16 + HPV52 were the most frequent in Zhejiang. Further study is necessary to explore the potential role of multiple HPV infections, especially the interactions among these common specific HPV genotypes in the development of cervical cancer.

To our knowledge, this study provides the largest scale information on the recent HPV prevalence and genotype distribution in Xiamen, and even in Fujian Province, China. However, several limitations existed. First, our study only included those women who underwent HPV testing in the gynecological outpatient and health examination center of Women and Children’s Hospital, School of Medicine, Xiamen University, and most of them were outpatients with gynecological disorders. In this respect, the findings may not represent all women in Xiamen, even in Fujian. Second, we did not collect detailed behavioral information such as the number of sexual partners, smoking habits, to assess the impact of these factors on the prevalence of HPV infection. Third, the results of cervical cytology or histology abnormalities were not collected, thus, we cannot further analysis the characteristics of HPV infection based on cervical lesion classification.

In conclusion, the age-specific prevalences of any HPV showed a bimodal distribution, with peaks at the women aged <25 years and 60–64 years, which provide evidence for the younger females for HPV vaccination and provide clinical guideline for perimenopausal women in cervical cancer screening in Xiamen. The prevalent genotypes were HPV52, 58, 16, 51, and 53, which suggests that more attention should be paid to these genotypes in vaccine development and cervical cancer screening process in Xiamen. In addition, we found the prevalence of HPV increased in 2022 and 2023, although it may be related to the free cervical cancer screening program implemented, it still deserves to pay closer attention and monitor the changes of HPV infection in the region in the future.

## Data availability statement

The original contributions presented in the study are included in the article/Supplementary material, further inquiries can be directed to the corresponding authors.

## Ethics statement

The studies involving humans were approved by Women and Children’s Hospital, School of Medicine, Xiamen Ethical Review Board. The studies were conducted in accordance with the local legislation and institutional requirements. Written informed consent from the patients was waived due to the retrospective nature of the study.

## Author contributions

XY: Data curation, Formal analysis, Funding acquisition, Investigation, Methodology, Project administration, Resources, Supervision, Visualization, Writing – original draft, Writing – review & editing. QL: Conceptualization, Data curation, Investigation, Resources, Writing – review & editing. YC: Formal analysis, Validation, Visualization, Writing – original draft. ZD: Data curation, Formal analysis, Writing – review & editing. YH: Data curation, Writing – review & editing. YiZ: Data curation, Validation, Writing – review & editing. JZ: Data curation, Validation, Writing – review & editing. WW: Data curation, Validation, Writing – review & editing. LZ: Data curation, Validation, Writing – review & editing. JX: Data curation, Validation, Writing – review & editing. CX: Data curation, Validation, Writing – review & editing. YG: Conceptualization, Data curation, Resources, Validation, Writing – review & editing. YuZ: Conceptualization, Funding acquisition, Resources, Supervision, Writing – review & editing.
