# Supplementary material for: Epidemiology of human papillomavirus infection in women from Xiamen, China, 2013 to 2023
Source: Front Public Health. 2024 Mar 21;12:1332696. doi: 10.3389/fpubh.2024.1332696 (PMC11000419; doi:10.3389/fpubh.2024.1332696)
Supplement: Supplementary file 1 [file Table_1.DOCX]

**Table S1. Prevalence of HPV infection by year.**

| year | N | Any HPV | | | HR-HPV | | | LR-HPV | | |
| --- | --- | --- | --- | --- | --- | --- | --- | --- | --- | --- |
|  |  | n | % | 95%CI | n | % | 95%CI | n | % | 95%CI |
| 2013 | 5608 | 1471 | 26.2 | 25.1, 27.4 | 1163 | 20.7 | 19.7, 21.8 | 504 | 9.0 | 8.3, 9.8 |
| 2014 | 6759 | 1507 | 22.3 | 21.3, 23.3 | 1193 | 17.6 | 16.7, 18.6 | 499 | 7.4 | 6.8, 8.1 |
| 2015 | 10809 | 2233 | 20.7 | 19.9, 21.4 | 1789 | 16.5 | 15.8, 17.2 | 762 | 7.0 | 6.5, 7.5 |
| 2016 | 13393 | 2748 | 20.5 | 19.8, 21.2 | 2160 | 16.1 | 15.5, 16.7 | 998 | 7.4 | 7.0, 7.9 |
| 2017 | 14341 | 2698 | 18.8 | 18.2, 19.5 | 2168 | 15.1 | 14.5, 15.7 | 863 | 6.0 | 5.6, 6.4 |
| 2018 | 18962 | 3606 | 19.0 | 18.5, 19.6 | 2892 | 15.2 | 14.6, 15.7 | 1136 | 5.9 | 5.6, 6.3 |
| 2019 | 25278 | 4736 | 18.7 | 18.3, 19.2 | 3913 | 15.5 | 15.0, 15.9 | 1367 | 5.4 | 5.1, 5.7 |
| 2020 | 19168 | 2840 | 14.8 | 14.3, 15.3 | 2328 | 12.2 | 11.7, 12.7 | 782 | 4.1 | 3.8, 4.4 |
| 2021 | 21076 | 3063 | 14.5 | 14.1, 15.0 | 2439 | 11.5 | 11.1, 12.0 | 939 | 4.4 | 4.1, 4.7 |
| 2022 | 16561 | 2945 | 17.8 | 17.2, 18.4 | 2299 | 13.5 | 13.0, 14.0 | 1151 | 6.7 | 6.3, 7.1 |
| 2023 | 7094 | 1351 | 19.0 | 18.1, 20.0 | 1077 | 14.4 | 13.6, 15.2 | 525 | 6.8 | 6.3, 7.4 |

Any HPV: Any of 21 detected HPV types; HR-HPV: high-risk HPV types (16, 18, 31, 33, 35, 39, 45, 51, 52, 56, 58, 59, and 68); LR-HPV: low-risk HPV types (6, 11, 42, 43, 44, 53, 66, and 81); HPV: human papillomavirus.

**Table S2. Prevalence of HPV infection in different age groups.**

| Age group, year | N | Any HPV | | | HR-HPV | | | LR-HPV | | |
| --- | --- | --- | --- | --- | --- | --- | --- | --- | --- | --- |
|  |  | n | % | 95%CI | n | % | 95%CI | n | % | 95%CI |
| <25 | 8347 | 2605 | 31.2 | 30.2, 32.2 | 2050 | 24.6 | 23.6, 25.5 | 1105 | 13.2 | 12.5, 14.0 |
| 25-29 | 33370 | 6159 | 18.5 | 18.0, 18.9 | 4907 | 14.7 | 14.3, 15.1 | 2053 | 6.2 | 5.9, 6.4 |
| 30-34 | 43835 | 6910 | 15.8 | 15.4, 16.1 | 5466 | 12.5 | 12.2, 12.8 | 2090 | 4.8 | 4.6, 5.0 |
| 35-39 | 27275 | 4465 | 16.4 | 15.9, 16.8 | 3543 | 13.0 | 12.6, 13.4 | 1342 | 4.9 | 4.7, 5.2 |
| 40-44 | 18080 | 3144 | 17.4 | 16.8, 18.0 | 2498 | 13.8 | 13.3, 14.3 | 921 | 5.1 | 4.8, 5.4 |
| 45-49 | 13831 | 2447 | 17.7 | 17.1, 18.3 | 1957 | 14.2 | 13.6, 14.7 | 729 | 5.3 | 4.9, 5.7 |
| 50-54 | 7901 | 1652 | 20.9 | 20.0, 21.8 | 1320 | 16.7 | 15.9, 17.6 | 546 | 6.9 | 6.4, 7.5 |
| 55-59 | 3536 | 972 | 27.5 | 26.0, 29.0 | 798 | 22.6 | 21.2, 24.0 | 331 | 9.4 | 8.4, 10.4 |
| 60-64 | 1579 | 520 | 32.9 | 30.6, 35.3 | 442 | 28.0 | 25.8, 30.3 | 166 | 10.5 | 9.0, 12.1 |
| ≥65 | 1295 | 324 | 25.0 | 22.7, 27.5 | 271 | 20.9 | 18.7, 23.3 | 116 | 9.0 | 7.5, 10.7 |

Any HPV: Any of 21 detected HPV types; HR-HPV: high-risk HPV types (16, 18, 31, 33, 35, 39, 45, 51, 52, 56, 58, 59, and 68); LR-HPV: low-risk HPV types (6, 11, 42, 43, 44, 53, 66, and 81); HPV: human papillomavirus.

**Table S3. Prevalence of HPV involved in women with HPV co-infection by genotypes.**

| HPV type | 16 | 18 | 31 | 33 | 35 | 39 | 45 | 51 | 52 | 56 | 58 | 59 | 68 | 6 | 11 | 42 | 43 | 44 | 53 | 66 | 81 |
| --- | --- | --- | --- | --- | --- | --- | --- | --- | --- | --- | --- | --- | --- | --- | --- | --- | --- | --- | --- | --- | --- |
| 16 | 2.489 | 0.088 | 0.056 | 0.062 | 0.014 | **0.102** | 0.020 | **0.125** | **0.238** | 0.043 | **0.180** | 0.033 | 0.066 | 0.079 | 0.054 | 0.023 | 0.018 | 0.020 | 0.098 | 0.053 | 0.084 |
| 18 | 0.088 | 1.098 | 0.025 | 0.034 | 0.008 | 0.053 | 0.008 | 0.051 | **0.114** | 0.026 | 0.079 | 0.025 | 0.028 | 0.035 | 0.030 | 0.015 | 0.009 | 0.008 | 0.048 | 0.027 | 0.053 |
| 31 | 0.056 | 0.025 | 0.668 | 0.025 | 0.003 | 0.030 | 0.008 | 0.031 | 0.058 | 0.014 | 0.051 | 0.017 | 0.019 | 0.020 | 0.021 | 0.006 | 0.003 | 0.008 | 0.031 | 0.023 | 0.030 |
| 33 | 0.062 | 0.034 | 0.025 | 0.831 | 0.008 | 0.043 | 0.006 | 0.040 | 0.076 | 0.020 | 0.076 | 0.022 | 0.030 | 0.021 | 0.019 | 0.006 | 0.006 | 0.009 | 0.048 | 0.024 | 0.049 |
| 35 | 0.014 | 0.008 | 0.003 | 0.008 | 0.209 | 0.011 | 0.003 | 0.011 | 0.020 | 0.006 | 0.009 | 0.006 | 0.005 | 0.004 | 0.003 | 0.003 | 0.004 | 0.003 | 0.016 | 0.009 | 0.018 |
| 39 | **0.102** | 0.053 | 0.030 | 0.043 | 0.011 | 1.683 | 0.015 | 0.086 | **0.170** | 0.039 | **0.109** | 0.027 | 0.051 | 0.045 | 0.033 | 0.028 | 0.013 | 0.025 | 0.087 | 0.043 | 0.083 |
| 45 | 0.020 | 0.008 | 0.008 | 0.006 | 0.003 | 0.015 | 0.227 | 0.014 | 0.024 | 0.010 | 0.018 | 0.006 | 0.009 | 0.012 | 0.010 | 0.002 | 0.001 | 0.003 | 0.010 | 0.008 | 0.011 |
| 51 | **0.125** | 0.051 | 0.031 | 0.040 | 0.011 | 0.086 | 0.014 | 1.826 | **0.191** | 0.048 | **0.107** | 0.031 | 0.063 | 0.056 | 0.032 | 0.052 | 0.016 | 0.018 | **0.103** | 0.038 | 0.093 |
| 52 | **0.238** | **0.114** | 0.058 | 0.076 | 0.020 | **0.170** | 0.024 | **0.191** | 3.997 | 0.086 | **0.239** | 0.057 | **0.118** | 0.084 | 0.056 | 0.045 | 0.019 | 0.041 | **0.206** | 0.075 | **0.205** |
| 56 | 0.043 | 0.026 | 0.014 | 0.020 | 0.006 | 0.039 | 0.010 | 0.048 | 0.086 | 0.669 | 0.064 | 0.016 | 0.031 | 0.021 | 0.011 | 0.012 | 0.009 | 0.011 | 0.050 | 0.023 | 0.040 |
| 58 | **0.180** | 0.079 | 0.051 | 0.076 | 0.009 | **0.109** | 0.018 | **0.107** | **0.239** | 0.064 | 2.576 | 0.041 | 0.067 | 0.076 | 0.038 | 0.030 | 0.020 | 0.020 | **0.122** | 0.062 | **0.112** |
| 59 | 0.033 | 0.025 | 0.017 | 0.022 | 0.006 | 0.027 | 0.006 | 0.031 | 0.057 | 0.016 | 0.041 | 0.458 | 0.013 | 0.020 | 0.019 | 0.008 | 0.006 | 0.007 | 0.040 | 0.020 | 0.036 |
| 68 | 0.066 | 0.028 | 0.019 | 0.030 | 0.005 | 0.051 | 0.009 | 0.063 | **0.118** | 0.031 | 0.067 | 0.013 | 1.029 | 0.025 | 0.013 | 0.014 | 0.006 | 0.014 | 0.062 | 0.025 | 0.055 |
| 6 | 0.079 | 0.035 | 0.020 | 0.021 | 0.004 | 0.045 | 0.012 | 0.056 | 0.084 | 0.021 | 0.076 | 0.020 | 0.025 | 0.761 | 0.022 | 0.009 | 0.009 | 0.007 | 0.036 | 0.036 | 0.045 |
| 11 | 0.054 | 0.030 | 0.021 | 0.019 | 0.003 | 0.033 | 0.010 | 0.032 | 0.056 | 0.011 | 0.038 | 0.019 | 0.013 | 0.022 | 0.557 | 0.005 | 0.006 | 0.004 | 0.023 | 0.017 | 0.022 |
| 42 | 0.023 | 0.015 | 0.006 | 0.006 | 0.003 | 0.028 | 0.002 | 0.052 | 0.045 | 0.012 | 0.030 | 0.008 | 0.014 | 0.009 | 0.005 | 0.390 | 0.011 | 0.009 | 0.017 | 0.007 | 0.027 |
| 43 | 0.018 | 0.009 | 0.003 | 0.006 | 0.004 | 0.013 | 0.001 | 0.016 | 0.019 | 0.009 | 0.020 | 0.006 | 0.006 | 0.009 | 0.006 | 0.011 | 0.227 | 0.008 | 0.016 | 0.009 | 0.010 |
| 44 | 0.020 | 0.008 | 0.008 | 0.009 | 0.003 | 0.025 | 0.003 | 0.018 | 0.041 | 0.011 | 0.020 | 0.007 | 0.014 | 0.007 | 0.004 | 0.009 | 0.008 | 0.438 | 0.022 | 0.007 | 0.025 |
| 53 | 0.098 | 0.048 | 0.031 | 0.048 | 0.016 | 0.087 | 0.010 | **0.103** | **0.206** | 0.050 | **0.122** | 0.040 | 0.062 | 0.036 | 0.023 | 0.017 | 0.016 | 0.022 | 1.743 | 0.041 | **0.099** |
| 66 | 0.053 | 0.027 | 0.023 | 0.024 | 0.009 | 0.043 | 0.008 | 0.038 | 0.075 | 0.023 | 0.062 | 0.020 | 0.025 | 0.036 | 0.017 | 0.007 | 0.009 | 0.007 | 0.041 | 0.721 | 0.044 |
| 81 | 0.084 | 0.053 | 0.030 | 0.049 | 0.018 | 0.083 | 0.011 | 0.093 | **0.205** | 0.040 | **0.112** | 0.036 | 0.055 | 0.045 | 0.022 | 0.027 | 0.010 | 0.025 | **0.099** | 0.044 | 1.608 |
